# The effect of one additional driver mutation on tumor progression

**DOI:** 10.1111/eva.12020

**Published:** 2012-12-10

**Authors:** Johannes G Reiter, Ivana Bozic, Benjamin Allen, Krishnendu Chatterjee, Martin A Nowak

**Affiliations:** 1 IST Austria (Institute of Science and Technology Austria)Klosterneuburg, Austria; 2 Program for Evolutionary Dynamics, Harvard UniversityCambridge, MA, USA; 3 Department of Mathematics, Harvard UniversityCambridge, MA, USA; 4 Department of Mathematics, Emmanuel CollegeBoston, MA, USA; 5 Department of Organismic and Evolutionary Biology, Harvard UniversityCambridge, MA, USA

**Keywords:** branching process, cancer, clonal expansion, density dependence, driver mutation, stochastic models

## Abstract

Tumor growth is caused by the acquisition of driver mutations, which enhance the net reproductive rate of cells. Driver mutations may increase cell division, reduce cell death, or allow cells to overcome density-limiting effects. We study the dynamics of tumor growth as one additional driver mutation is acquired. Our models are based on two-type branching processes that terminate in either tumor disappearance or tumor detection. In our first model, both cell types grow exponentially, with a faster rate for cells carrying the additional driver. We find that the additional driver mutation does not affect the survival probability of the lesion, but can substantially reduce the time to reach the detectable size if the lesion is slow growing. In our second model, cells lacking the additional driver cannot exceed a fixed carrying capacity, due to density limitations. In this case, the time to detection depends strongly on this carrying capacity. Our model provides a quantitative framework for studying tumor dynamics during different stages of progression. We observe that early, small lesions need additional drivers, while late stage metastases are only marginally affected by them. These results help to explain why additional driver mutations are typically not detected in fast-growing metastases.

## Introduction

Disease progression in cancer is driven by somatic evolution of cells (Nordling 1953; Nowell 1976; Vogelstein and Kinzler 1993; Hanahan and Weinberg 2000; Vogelstein and Kinzler 2004; Merlo *et al*. 2006; Gatenby and Gillies 2008). Mathematical modeling (Wodarz and Komarova 2005) can provide quantitative insights into many aspects of this process, including the age incidence of cancer (Armitage and Doll 1954; Knudson 1971, 2001; Luebeck and Moolgavkar 2002; Michor *et al*. 2006; Meza *et al*. 2008), the role of genetic instability in tumor progression (Nowak *et al*. 2002; Komarova *et al*. 2002, 2003; Michor *et al*. 2003; Rajagopalan *et al*. 2003; Michor *et al*. 2005b; Nowak *et al*. 2006), the timing of disease progression events (Moolgavkar and Knudson 1981; Nowak *et al*. 2003; Iwasa *et al*. 2004, 2005; Beerenwinkel *et al*. 2007; Jones *et al*. 2008a; Attolini *et al*. 2010; Bozic *et al*. 2010; Durrett and Moseley 2010; Gerstung and Beerenwinkel 2010; Yachida *et al*. 2010; Durrett and Mayberry 2011; Gerstung *et al*. 2011; Martens *et al*. 2011), the evolution of resistance to chemotherapy (Coldman and Goldie 1985, 1986; Goldie and Coldman 1986, 1998), the dynamics of targeted cancer therapy (Michor *et al*. 2005a; Dingli and Michor 2006; Leder *et al*. 2011; Bozic *et al*. 2012; Diaz *et al*. 2012), and genetic heterogeneity within tumors (Durrett *et al*. 2011; Iwasa and Michor 2011).

Tumors are initiated by a genetic event that provides a previously normal cell with an increased reproductive rate (a fitness advantage) compared with surrounding cells. In the case of colon cancer, this initiating event (usually inactivation of the *APC* tumor suppressor gene) starts the growth of a micro-adenoma (Kinzler and Vogelstein 1996). Subsequent genetic alterations can further increase the reproductive potential of tumor cells and lead to the development of a large adenoma and carcinoma (Vogelstein *et al*. 1988; Baker *et al*. 1989; Fearon and Vogelstein 1990). Metastasis, the dissemination and growth of tumor cells in distant organs, is thought to occur late in the course of tumor evolution (Yachida *et al*. 2010). Few, if any, selective events are required to transform a highly invasive cancer cell into one with the capacity to metastasize (Jones *et al*. 2008a).

Here, we study how one additional driver mutation affects tumor growth. We model a stochastically growing lesion and explore the consequence of an additional driver mutation, which might appear. Driver mutations are defined as those that increase the fitness of tumor cells and contribute to the carcinogenic process (Frank and Nowak 2004; Maley *et al*. 2004; Sjöblom *et al*. 2006; Greenman *et al*. 2007; Wood *et al*. 2007; Jones *et al*. 2008b; Parsons *et al*. 2008). In cancer biology, the fitness of a cell represents its reproductive potential. Many different mechanisms can increase the net growth rate of cancerous cells such as sustaining proliferative signaling, evading growth suppressors, resisting cell death, or gaining unlimited replicative potential (Hanahan and Weinberg 2011). Driver mutations constitute only a fraction of the genetic alterations found in tumor cells; the remainder are ‘passengers’, which do not alter fitness but hitchhike to high frequency on the basis of driver mutations. Bozic *et al*. (2010) give a formula for the predicted relationship between the numbers of driver and passenger mutations acquired over time.

We model tumor growth using a discrete-time branching process (also known as the Galton–Watson process) (Athreya and Ney 1972). We consider two types of cells: resident (type 0) and mutant (type 1) cells. Mutant cells have one additional driver mutation with respect to resident cells. Thus, our model could be thought of as a one-bit description of tumor dynamics, where one bit encodes the genotype of a cell with respect to the additional driver mutation. This model is a generalization of the Luria-Delbrück model used in studying bacterial evolution (Luria and Delbrück 1943; Zheng 1999; Dewanji *et al*. 2005). Similar two-type stochastic models of cancer evolution were previously used to study the evolution of resistance to cancer therapy (Goldie and Coldman 1979; Coldman and Goldie 1983; Komarova and Wodarz 2005; Iwasa *et al*. 2006; Foo and Michor 2010; Bozic *et al*. 2012; Diaz *et al*. 2012) and stochastic dynamics in healthy and preneoplastic tissue (Clayton *et al*. 2007; Klein *et al*. 2010; Antal and Krapivsky 2011).

Our model can be applied to different stages of tumor progression. For example, the additional driver mutation could be the mutation activating the *KRAS*/*BRAF* pathway in a small colorectal adenoma, associated with the transformation from small to large adenoma, or the mutation that transforms benign adenoma into infiltrating carcinoma (Jones *et al*. 2008a). Finally, the additional driver mutation can be a new driver in a metastatic lesion. We are particularly interested in the following question: under which conditions does an additional driver mutation accelerate tumor progression?

## Materials and methods

### The model

**Figure 1:**
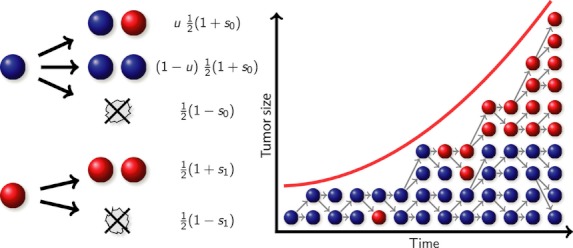
Illustration of the branching process. A tumor is initiated with a single resident cell. At each time step, each cell either divides or dies, leading to a stochastically growing tumor. Resident cells (blue) have a division probability of 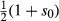, while mutant cells (red) have a division probability of 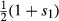. Additionally, resident cells may mutate upon division, with probability *u*.

We model tumor evolution as a discrete-time, two-type branching process. At each time step, each cell either divides (yielding two daughter cells) or dies. These events occur independently for each cell. Each resident cell divides with probability 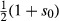 and dies with probability 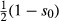. Here, 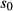 denotes the growth coefficient (which we define as division probability minus death probability per time step) of resident cells, and may be constant or variable depending on the model under consideration (see below). Similarly, mutant cells divide with probability 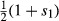 and die with probability 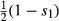. When a resident cell divides, one of the two daughter cells can receive an additional driver mutation (making it a mutant) with probability *u* (Fig. 1). This parameter *u* reflects both the point mutation rate in resident cells and the number of positions in the genome that can give rise to the next driver mutation. Each time step in our model corresponds to the time between divisions of a typical tumor cell. We assume that the time between cell divisions is the same for resident and mutant cells. Tumor progression is initiated by a single resident cell. We study the dynamics of tumor progression by considering two possible endpoints: (i) extinction of the tumor and (ii) the tumor reaches a certain size, *M* (which might correspond to clinical detection).

We study two related models that differ in the growth dynamics of the resident cells.

In the *exponential growth model*, the growth coefficients 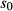 and 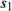 are constant, so that both resident and mutant cells grow exponentially on average. Moreover, mutant cells have a growth advantage compared with resident cells (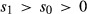) and can therefore potentially accelerate tumor progression. This model can be viewed as a special case of the model used in Bozic *et al*. 2010, in which multiple driver mutations can occur in sequence.

In the *logistic growth model*, resident cells are constrained by a density limit. They grow exponentially at first, but eventually reach a steady state around a certain number of cells (the carrying capacity *K*). For our stochastic model, this means that the division probability of the resident cells varies with tumor size. We achieve this by considering a variable growth coefficient 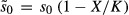, where *X* is the current size of the tumor. In this case, the constant 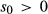 represents only the initial growth coefficient of resident cells (when *X*≪*K*), while the variable 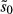 represents the growth coefficient at any tumor size *X*. For tumor sizes *X* for which division probability of resident cells would fall below 0 (or for which, equivalently, 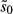 would fall below −1), we set 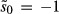. Mutant cells have no density limit, but rather have a constant growth coefficient 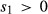. This logistic growth model describes the situation where additional mutations are needed for the tumor to overcome current geometric and metabolic constraints (Spratt *et al*. 1993; Jiang *et al*. 2005). Density-dependent branching process models have previously been used by Tan (1986) to model tumor growth and Bozic *et al*. (2012) to model acquired resistance to targeted therapy.

### Simulations

We use computer simulations to understand the evolutionary dynamics of our stochastic model of tumor progression. To ensure an efficient processing of the discrete-time Galton–Watson branching process, we only store the number of resident and mutant cells in each time step. By sampling from a multinomial distribution, we obtain the number of cells of both types in the next generation (Bozic *et al*. 2010). Note that in the logistic growth model, the birth probabilities for the resident cells depend on the size of the tumor, and therefore, we need to calculate them in each generation.

**Figure 2:**
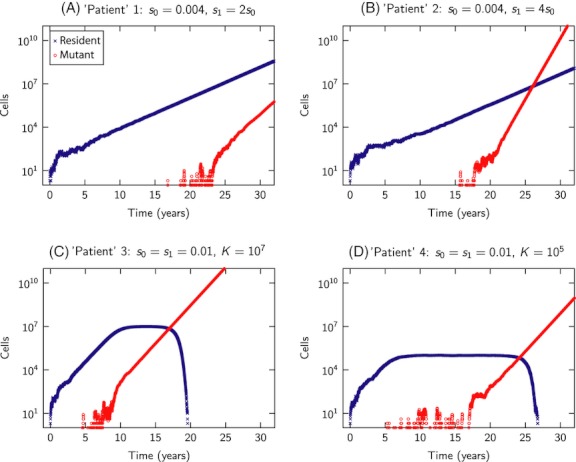
Driver mutation effect on tumor progression under various conditions. These plots show typical simulation results for the exponential growth model (A and B) and the logistic growth model (C and D). A higher growth coefficient of the mutant type (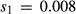 in A vs 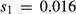 in B) increases its survival probability and reduces the time until the mutant type becomes dominant. In C and D, the additional driver mutation is neutral (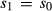). The resident cells decline at the point when the mutant cells (and hence the total number of cells) exceed the carrying capacity of the resident cells. In C, we have *αKu* > 1; thus, the mutant type arises while the resident type is still expanding (see “Logistic growth model” subsection of Results). In D, we have *αKu* < 1 and hence the resident population remains at carrying capacity for a significant period of time before the mutant type arises. Parameter values: driver mutation rate 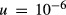, average cell division time is 3 days.

In Fig. 2 A and B, we show two realizations of the exponential growth model, corresponding to tumor evolution in two ‘patients’. Similarly, in Fig. 2 C and D, we show two examples of tumor evolution in the logistic growth model.

### Parameter selection

The effects of additional driver mutations depend on the driver mutation rate. This rate is the product of the number of positions in the genome that would lead to a new driver mutation if altered and the point mutation rate. The point mutation rate in normal and cancer tissues has been reported to be in the range 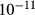 to 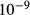 (Albertini *et al*. 1990; Cervantes *et al*. 2002; Jones *et al*. 2008a). It was estimated that there are ∼30 000 positions in the genome that could become driver mutations (Bozic *et al*. 2010). If any of them could become the next driver in the tumor, then the driver mutation rate *u* would be on the order of 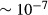 to 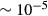, and if only a subset of all driver mutations could become the next driver, the driver mutation rate would be much smaller. Some types of genetic instability could additionally increase the point mutation rate (Thibodeau *et al*. 1993; Loeb 1994; Lengauer *et al*. 1998). To account for all these possibilities, we will consider driver mutation rates in the range 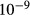 to 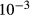. We are assuming that tumors we are modeling have already evolved chromosomal instability (CIN) and therefore that inactivation of a single copy of a tumor suppressor gene leads to a new driver mutation, as the other copy will be lost soon thereafter (Nowak *et al*. 2002, 2004).

Time between cell divisions has been reported to be on average 4 days in colorectal cancer (Jones *et al*. 2008a) and 3 days in glioblastoma multiforme (Hoshino and Wilson 1979). In this paper, we will assume the time between cell divisions is 3 days.

Growth rates of tumors can be estimated from the reports of the tumor volume doubling time. Average reported volume doubling times of breast cancer range between 105 and 270 days (Kusama *et al*. 1972; Amerlöv *et al*. 1992) and between 61 and 269 days for adenocarcinoma of the lung (Schwartz 1961; Spratt *et al*. 1963; Weiss 1974). It follows that the average growth coefficient of these advanced tumors can vary from 0.008 to 0.035, assuming 3 days between cell divisions. Early lesions have smaller growth rates, and some metastases can grow even faster (Friberg and Mattson 1997). For this reason, in our paper we consider growth coefficients of resident cells from 0.002 to 0.04.

At some size during tumor growth, the tumor needs to develop blood vessels to provide enough oxygen and nutrients required for survival and further growth to the tumor cells. It has been estimated that the maximum size of a tumor without blood vessels is 1-2 mm in diameter (Kerbel 2000). This maximum size acts as a carrying capacity in tumor progression. Based on the prior estimation, this carrying capacity is on the order of millions of cells in our logistic growth model. In our simulations, we will consider carrying capacities of 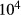 to 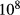 cells.

## Results

Our first result applies to either version of the model. We find that, for reasonably small mutation rate *u* (and reasonably large density limit *K* in the logistic model), the additional driver mutation has no effect on the overall survival probability of the tumor. This is because the mutation generally occurs when the number of cells in the tumor is ∼1/*u* (or *K*, in the logistic model when *K* < 1/*u*) and there is no longer a chance for extinction.

Following Bozic *et al*. (2010), we obtain that for either version, the tumor survival probability is given by 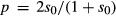. This is the probability that a lineage arising from a single cell will not become extinct. When the growth coefficient of resident cells is small (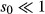), this survival probability can be approximated as 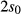.

### Exponential growth model

We now focus on the basic model of exponential tumor growth, assuming small growth coefficients of resident and mutant cells and a small driver mutation rate (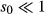, 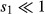, and 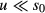). Following Bozic *et al*. (2010), we calculate the expected number of resident cells at time *t* (measured in units of cell division time) as


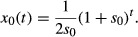
(1)

We note that this average is conditioned on the survival of the tumor. The expected time until the appearance of the first mutant cell with a surviving lineage is


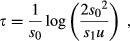
(2)

assuming a small growth coefficient of resident and mutant cells and small mutation rate (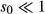, 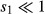, and 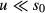). The expected number of mutant cells *t*′ time steps after the appearance of the first mutant cell with a surviving lineage is


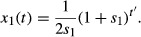
(3)

Although there is stochasticity in the timing of the appearance of the first mutant cell with a surviving lineage, we can achieve a good approximation to the number of mutant cells at time *t* by setting *t*′ = *t*−*τ* in eqn (3).

Using the eqn (1) for the average number of resident cells, we can approximate the time until there are *M* resident cells in the tumor as


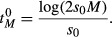
(4)

Respectively, the time 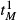 until there are *M* mutant cells in the tumor is:


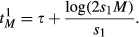
(5)

We note that in eqns (1), (4), and (5), time is measured in numbers of cell divisions and needs to be multiplied by average time between cell divisions to represent real time.

**Figure 3:**
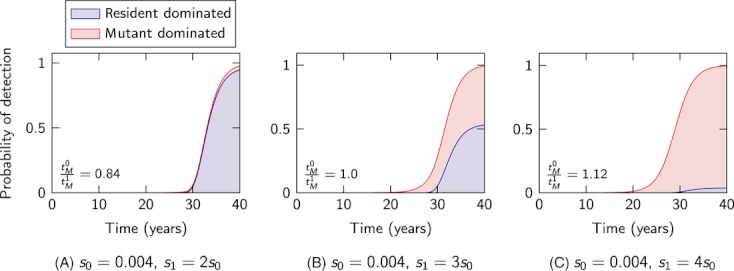
Dominating cell types in the tumor at detection time. Cumulative probability distribution of the tumor detection time (i.e., 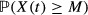), as calculated from 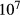 simulation runs. The blue shaded regions correspond to tumors dominated by resident cells (more than 50% of the tumor cells at detection time are resident), while the red shaded regions correspond to tumors dominated by mutant cells at detection time (more than 50% of the tumor cells at detection time are mutants). The tumor composition at detection time can be estimated by the ratio 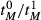. Parameter values: driver mutation rate 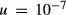, detection size 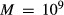 cells, average cell division time is 3 days.

Since resident and mutant cells grow exponentially with different growth rates, we expect that tumors will most often be dominated by one cell type: for short times, tumors will consist mostly of resident cells; for long times, they will consist mostly of mutant cells (Fig. 3). Thus, we approximate the expected detection time of the tumor as


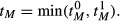
(6)

**Figure 4:**
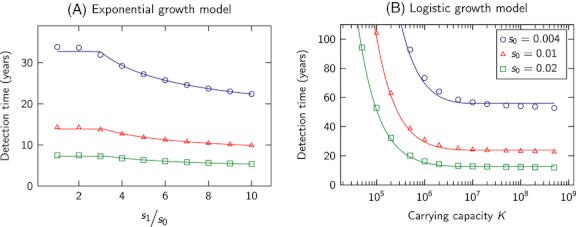
Comparison of analytical and simulation results for the expected time of tumor detection. Markers (circle, triangle, square) indicate simulation results while curves represent analytic predictions. In the exponential model (A), we observe that, for typical mutation rates, the additional driver needs to have a three times higher growth coefficient in order for the mutant type to accelerate tumor progression prior to detection. In the logistic growth model (B), the additional driver mutation is neutral (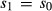). We see that small carrying capacities (with *αKu* < 1) significantly slow tumor progression, while large carrying capacities (*αKu* > 1) have little effect. Simulation results are averages over 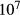 runs. Parameter values: detection size 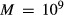 cells, driver mutation rate 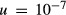, average cell division time is 3 days.

Figure 4A shows the agreement between formula (6) and computer simulations.

If 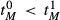, we expect that the tumor will consist mostly of resident cells (Fig. 3). Consequently, the additional driver mutation does not have a significant effect on detection time. This observation suggests the following approximate rule: the additional driver mutation has an effect if


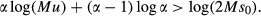
(7)

Here, *α* denotes the ratio 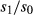. From the above inequality, we see that larger *α* and *u* increase the likelihood of a mutant-dominated tumor (at the time of detection), while larger 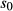 increases the chance of a resident-dominated tumor.

In many clinical contexts, it is reasonable to assume that *Mu* ≫ 1 (see Discussion). In this case, the above rule can be simplified further by rewriting the left-hand side of inequality (7) and observing that *α* log (*αMu*) ≫ log *α*. This leads to the following simplification of inequality (7):


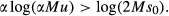
(8)

**Figure 5:**
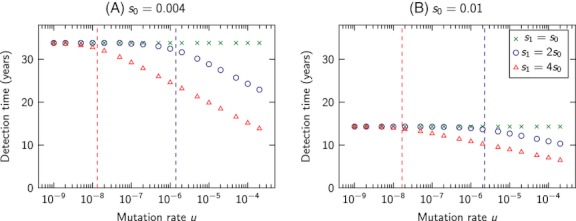
Effect of the additional driver mutation on tumor detection time. The markers represent simulation results for the exponential growth model, with mutant growth coefficient equal to (green crosses), twice (blue circles), and four times (red triangles) the resident growth coefficient. The dashed lines correspond to the threshold (8) indicating when the additional driver mutation accelerates tumor progression. Simulation results are averages over 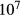 runs. Parameter values: detection size 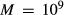 cells, average cell division time is 3 days.

We show the agreement between the rule (8) and simulations in Fig. 5. Using eqn (8) we find that, if the driver mutation rate is 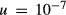, the detection size is 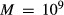, and the growth coefficient of resident cells is 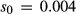, then mutant cells need a three times higher growth coefficient than resident cells to affect tumor detection time. For 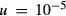 and the other parameter values are the same as before, mutant cells need a 1.6 times higher growth coefficient than resident cells to affect detection time.

### Logistic growth model

We now consider the situation where the growth of resident cells is density limited. To analyze the expected appearance time *τ* of the first mutant cell with surviving lineage, we need to distinguish between two cases: (i) the first surviving mutant is generated before the resident cells reach their carrying capacity and (ii) the first surviving mutant is generated when the resident cells are at their carrying capacity. In Appendix 1 A, we show that the first case is expected to occur for 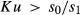 (or, equivalently, *αKu* > 1) and the second case for 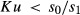 (*αKu* < 1). We note that for 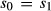, these two cases are divided by *Ku* = 1.

The expected appearance time *τ* of the first mutant cell with a surviving lineage is calculated in Appendix 1 A as


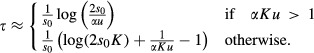
(9)

If the detection size *M* is smaller than the carrying capacity of resident cells, *K*, then the model behaves similarly to the exponential growth model. Thus, we restrict our attention to the case *K* < *M*. In that case, the expected detection time of the tumor is


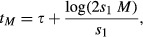
(10)

where *τ* is given by eqn (9). We show the excellent agreement between eqn (10) and simulation results in Fig. 4B. Using formula (10), we find that, for carrying capacity 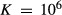, driver mutation rate 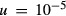, growth coefficients of resident and mutant cells 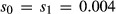, and detection size 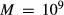 cells, the average tumor detection time is ∼46 years. For driver mutation rate 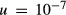 and all other parameters are the same as before, the average tumor detection time is ∼70 years. In any one patient, multiple such lesions could be seeded, but only a small fraction of them would reach detectable size in the lifetime of the patient (see Table 3). Additional results are provided in Appendix S.1.

## Discussion

Our results describe how additional driver mutations affect the dynamics of tumor growth in different stages of disease progression. Early lesions often have a limited growth potential due to spatial or metabolic constraints and need additional driver mutations to reach a detectable size. In contrast, many metastases exhibit fast exponential growth, which does not leave enough time for a new driver mutation to appear and reach significant abundance to affect detection time. In addition, metastases often have shorter doubling times (and thus larger 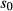) compared with early lesions (Welin *et al*. 1963; Tanaka *et al*. 2004). Thus, additional drivers can more significantly increase the growth rate (leading to a higher 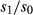 ratio) of an early lesion compared to a metastasis. An additional driver would have to increase an already large growth rate of a metastasis drastically to have an effect on detection time (Table 1). These results explain why metastases may not contain additional driver mutations compared to primary tumors (Jones *et al*. 2008a).

**Table 1:** Probability of tumor detection over time in the exponential growth model

		Probability of detection after					
		
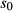	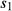	5 years	10 years	20 years	30 years	40 years	50 years
0.002	0.002	0.0	0.0	0.0	0.0	0.0	0.0
	0.004	0.0	0.0	0.0	0.0	0.0	0.003
	0.008	0.0	0.0	0.0	0.002	0.024	0.226
0.004	0.004	0.0	0.0	0.0	0.026	0.973	1.0
	0.008	0.0	0.0	0.001	0.436	0.974	1.0
	0.016	0.0	0.0	0.01	0.598	0.995	1.0
0.01	0.01	0.0	0.0	0.999	1.0	1.0	1.0
	0.02	0.0	0.002	1.0	1.0	1.0	1.0
	0.04	0.0	0.032	1.0	1.0	1.0	1.0
0.02	0.02	0.0	0.999	1.0	1.0	1.0	1.0
	0.04	0.0	0.999	1.0	1.0	1.0	1.0
	0.08	0.012	1.0	1.0	1.0	1.0	1.0
0.04	0.04	0.997	1.0	1.0	1.0	1.0	1.0
	0.08	0.997	1.0	1.0	1.0	1.0	1.0
	0.16	0.999	1.0	1.0	1.0	1.0	1.0

The birth probability of the resident and mutant cells is given by 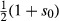 and 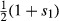, respectively. A higher growth coefficient of the mutant, 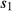, can accelerate tumor progression. When 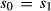, the detection time is independent of the mutation rate. The simulation results are averages over 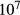 runs. Parameter values: detection size 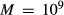 cells, driver mutation rate 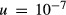, average cell division time is 3 days. (The value 0.0 corresponds to a probability below 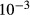.)

In the case that resident tumor cells are subject to a density limitation, the effect of this density limitation on tumor dynamics depends strongly on the product of the carrying capacity *K* and the driver mutation rate *u*. (More specifically, the dynamics depend on the product *αKu*, but 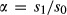 can be expected to be in the range 1–10, whereas *K* and *u* may be much more variable across different clinical contexts.) If 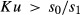, the first surviving mutant appears before the tumor growth is decelerated by the carrying capacity for the resident cells (see eqn (9) and Table 2); thus, the density constraint has little or no effect on the dynamics. However, if 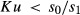, the first surviving mutant appears only after the tumor has reached the carrying capacity. In this case, the carrying capacity can tremendously decelerate tumor progression (Table 3). For example, if 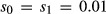 and 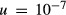, then for 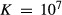 the expected detection time, 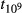, is 23.9 years but for 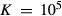, 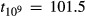 years. From these two examples, we see that in many cases, carrying capacities are either overcome almost as soon as they are reached (if 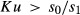) or delay tumors to such an extent that they never reach detectable size (if 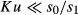). Only in the intermediate case that *Ku* has the same order of magnitude as 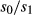 (which itself is likely in the range 0.1–1), would the delay in cancer progression due to carrying capacity be observable.

**Table 2:** Probability of tumor detection over time in the logistic growth model

		Probability of detection after					
		
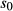	*K*	10 years	20 years	30 years	40 years	50 years	60 years
0.002	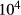	0.0	0.0	0.0	0.0	0.0	0.0
	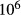	0.0	0.0	0.0	0.0	0.0	0.0
	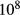	0.0	0.0	0.0	0.0	0.0	0.0
0.004	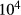	0.0	0.0	0.0	0.030	0.27	0.538
	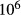	0.0	0.0	0.0	0.062	0.944	1.0
	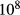	0.0	0.0	0.0	0.123	0.98	1.0
0.01	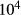	0.0	0.152	0.729	0.918	0.975	0.992
	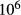	0.0	0.706	1.0	1.0	1.0	1.0
	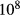	0.0	0.877	1.0	1.0	1.0	1.0
0.02	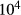	0.076	0.904	0.991	0.999	1.0	1.0
	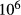	0.301	1.0	1.0	1.0	1.0	1.0
	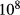	0.566	1.0	1.0	1.0	1.0	1.0
0.04	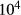	0.883	0.999	1.0	1.0	1.0	1.0
	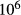	1.0	1.0	1.0	1.0	1.0	1.0
	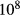	1.0	1.0	1.0	1.0	1.0	1.0

The resident cells have a birth probability of 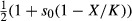, which depends on the current tumor size *X*. The birth probability of the mutant cells is constant 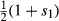. If the carrying capacity *K* is low but the mutation rate *u* is high (more precisely, if 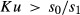), tumor progression is not decelerated. The simulation results are averages over 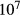 runs. Parameter values: growth coefficient 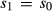, driver mutation rate 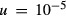 (see Table 3 for 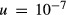), detection size 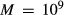 cells, average cell division time is 3 days. (The value 0.0 corresponds to a probability below 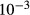.)

The product *Ku*—and more generally, products of the form (number of cells) × (mutation rate)—also plays an important role in quantifying the likelihood of treatment failure due to acquired resistance (Goldie and Coldman 1979; Coldman and Goldie 1983, 1986; Komarova and Wodarz 2005; Iwasa *et al*. 2006; Durrett and Moseley 2010; Foo and Michor 2010; Leder *et al*. 2011; Read *et al*. 2011; Bozic *et al*. 2012). Intuitively, this product represents the number of mutations generated per cell division time in a population of cells. If this product is much greater than one, then mutations of interest (e.g., driver mutations, resistance mutations) are ubiquitous; if the product is much less than one, then they are rare. This product can therefore be used as a rule of thumb to determine the danger posed by a certain variety of mutation. We caution, however, that other parameters, such as division rates, death rates, and time spent at a certain population size (Bozic *et al*. 2012), also play important roles in determining the likelihood of clinically relevant mutations.

**Table 3:** Probability of tumor detection over time in the logistic growth model

		Probability of detection after					
		
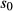	*K*	10 years	20 years	30 years	40 years	50 years	60 years
0.002	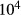	0.0	0.0	0.0	0.0	0.0	0.0
	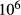	0.0	0.0	0.0	0.0	0.0	0.0
	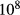	0.0	0.0	0.0	0.0	0.0	0.0
0.004	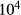	0.0	0.0	0.0	0.0	0.003	0.008
	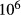	0.0	0.0	0.0	0.0	0.036	0.286
	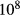	0.0	0.0	0.0	0.0	0.087	0.957
0.01	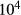	0.0	0.002	0.013	0.025	0.036	0.048
	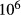	0.0	0.008	0.575	0.873	0.962	0.989
	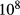	0.0	0.01	1.0	1.0	1.0	1.0
0.02	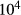	0.001	0.023	0.046	0.069	0.091	0.112
	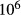	0.002	0.849	0.986	0.999	1.0	1.0
	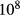	0.002	1.0	1.0	1.0	1.0	1.0
0.04	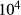	0.021	0.065	0.108	0.149	0.188	0.225
	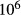	0.817	0.998	1.0	1.0	1.0	1.0
	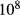	1.0	1.0	1.0	1.0	1.0	1.0

The resident cells have a birth probability of 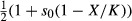, which depends on the current tumor size *X*. The birth probability of the mutant cells is constant 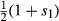. If the carrying capacity *K* and/or mutation rate *u* is small (more precisely, if 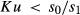), the tumor progression is significantly slowed by the density limitation. The simulation results are averages over 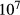 runs. Parameter values: growth coefficient 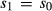, driver mutation rate 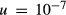 (see Table 2 for 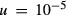), detection size 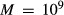 cells, average cell division time is 3 days. (The value 0.0 corresponds to a probability below 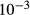.)

In this work, we derived conditions that help determine whether the additional driver mutation will significantly accelerate tumor progression. In reality, most solid tumors need several driver mutations to reach advanced carcinoma and metastatic stage that are most detrimental to the patient. When comparing our results to previous modeling work on the accumulation of multiple driver mutations in tumors (Beerenwinkel *et al*. 2007; Beckman 2009; Bozic *et al*. 2010), one should keep in mind that the times to detection of a lesion might be shorter than reported here if the cells could quickly receive several drivers.

## Figures and Tables

**Figure A.1:**
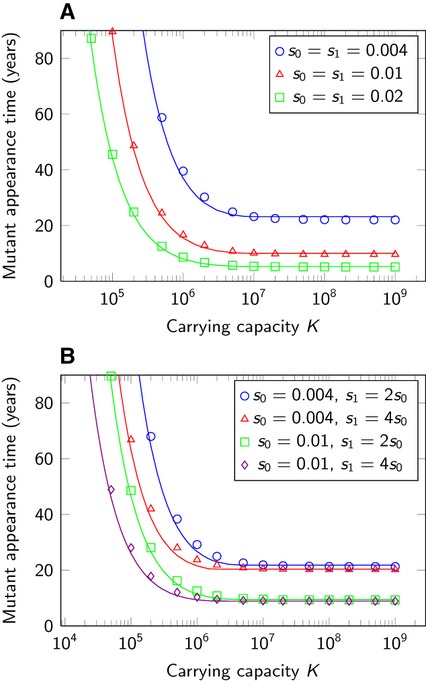
Mutant appearance time in the logistic growth model. Comparison of analytical and simulation results for the expected appearance time of the first surviving mutant in the logistic growth model. Circles, triangles, squares, and diamonds correspond to the average results of the simulation, and lines correspond to the analytical result, eqn (9). Simulation results are averages over 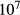 runs. Parameter values: detection size 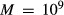 cells, driver mutation rate 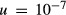, average cell division time is 3 days.

## Appendix A - Mutant appearance time

To analyze the expected appearance time *τ* of the first surviving mutant cell, we use the fact that until very close to their carrying capacity, resident cells will on average grow exponentially. Thus, we can approximate their growth by two phases: exponential and steady state. In this approximation, the average time to reach steady state is given by

(A.1)

If the first surviving mutant is generated during the exponential phase, then its average time of appearance *τ* is given by equation (2), and we must have . Comparing (2) and (A.1), we see that  is equivalent to . We conclude that (i) if  the first surviving mutant is generated before resident cells reach the carrying capacity and (ii) if  the first surviving mutant is generated after resident cells have reached the carrying capacity.

In the second case, we approximate the time until the appearance of the first surviving mutant, *τ*, by the time when the total expected number of surviving mutants produced reaches 1. This leads to the equation

(A.2)

Using the formula for a geometric series, we obtain

Substituting this in (A.2) and solving for *τ* yields

Using  and *K*≫1, we can approximate

## Supporting Information

**Appendix S1.** Supplementary results.
