# Supplementary material for: The effect of one additional driver mutation on tumor progression
Source: Evol Appl. 2012 Dec 10;6(1):34–45. doi: 10.1111/eva.12020 (PMC3567469; doi:10.1111/eva.12020)
Supplement: Supplementary file 1 [file eva0006-0034-SD1.pdf]

Appendix to

The effect of one additional driver mutation on tumor progression

Johannes G. Reiter, Ivana Bozic, Benjamin Allen, Krishnendu Chatterjee & Martin A. Nowak

S1 Supplementary results

		Probability of detection after					
s_0	s_1	5 years	10 years	20 years	30 years	40 years	50 years
0.002	0.002	0.0	0.0	0.0	0.0	0.0	0.0
	0.004	0.0	0.0	0.0	0.0	0.0	0.0
	0.008	0.0	0.0	0.0	0.0	0.0	0.003
0.004	0.004	0.0	0.0	0.0	0.026	0.973	1.0
	0.008	0.0	0.0	0.0	0.026	0.973	1.0
	0.016	0.0	0.0	0.0	0.04	0.974	1.0
0.01	0.01	0.0	0.0	0.999	1.0	1.0	1.0
	0.02	0.0	0.0	0.999	1.0	1.0	1.0
	0.04	0.0	0.0	1.0	1.0	1.0	1.0
0.02	0.02	0.0	0.999	1.0	1.0	1.0	1.0
	0.04	0.0	0.999	1.0	1.0	1.0	1.0
	0.08	0.0	0.999	1.0	1.0	1.0	1.0
0.04	0.04	0.997	1.0	1.0	1.0	1.0	1.0
	0.08	0.997	1.0	1.0	1.0	1.0	1.0
	0.16	0.997	1.0	1.0	1.0	1.0	1.0

Table S1: Probability of tumor detection over time in the exponential growth model when there are only a few drivers. The birth probability of the resident and mutant cells are given by $\frac{1}{2}(1+s_0)$ and $\frac{1}{2}(1+s_1)$, respectively. A higher growth coefficient of the mutant, s_1, can accelerate tumor progression. When $s_0 = s_1$, the detection time is independent of the mutation rate. The simulation results are averages over 10^7 runs. Parameter values: detection size $M = 10^9$ cells, driver mutation rate $u = 10^{-9}$, average cell division time is 3 days. (The value 0.0 corresponds to a probability below 10^{-3}.)

		Probability of detection after					
s_0	s_1	5 years	10 years	20 years	30 years	40 years	50 years
	0.002	0.0	0.0	0.0	0.0	0.0	0.0
0.002	0.004	0.0	0.0	0.0	0.0	0.022	0.283
	0.008	0.0	0.0	0.009	0.176	0.738	0.972
	0.004	0.0	0.0	0.0	0.026	0.973	1.0
0.004	0.008	0.0	0.0	0.007	0.661	0.997	1.0
	0.016	0.0	0.003	0.521	0.994	1.0	1.0
	0.01	0.0	0.0	0.999	1.0	1.0	1.0
0.01	0.02	0.0	0.024	1.0	1.0	1.0	1.0
	0.04	0.006	0.056	1.0	1.0	1.0	1.0
	0.02	0.0	0.999	1.0	1.0	1.0	1.0
0.02	0.04	0.007	1.0	1.0	1.0	1.0	1.0
	0.08	0.563	1.0	1.0	1.0	1.0	1.0
	0.04	0.997	1.0	1.0	1.0	1.0	1.0
0.04	0.08	0.999	1.0	1.0	1.0	1.0	1.0
	0.16	1.0	1.0	1.0	1.0	1.0	1.0

Table S2: Probability of tumor detection over time in the exponential growth model. The birth probability of the resident and mutant cells are given by $\frac{1}{2}(1+s_0)$ and $\frac{1}{2}(1+s_1)$, respectively. A higher growth coefficient of the mutant, s_1, can accelerate tumor progression. When $s_0 = s_1$, the detection time is independent of the mutation rate. The simulation results are averages over 10^7 runs. Parameter values: detection size $M = 10^9$ cells, driver mutation rate $u = 10^{-5}$, average cell division time is 3 days. (The value 0.0 corresponds to a probability below 10^{-3}.)

		Probability of detection after					
s_0	s_1	5 years	10 years	20 years	30 years	40 years	50 years
	0.002	0.0	0.0	0.0	0.0	0.0	0.0
0.002	0.004	0.0	0.0	0.0	0.002	0.840	0.996
	0.008	0.0	0.0	0.508	0.996	1.0	1.0
	0.004	0.0	0.0	0.0	0.026	0.973	1.0
0.004	0.008	0.0	0.0	0.543	0.999	1.0	1.0
	0.016	0.0	0.215	0.999	1.0	1.0	1.0
	0.01	0.0	0.0	0.999	1.0	1.0	1.0
0.01	0.02	0.0	0.835	1.0	1.0	1.0	1.0
	0.04	0.41	1.0	1.0	1.0	1.0	1.0
	0.02	0.0	0.999	1.0	1.0	1.0	1.0
0.02	0.04	0.544	1.0	1.0	1.0	1.0	1.0
	0.08	0.997	1.0	1.0	1.0	1.0	1.0
	0.04	0.997	1.0	1.0	1.0	1.0	1.0
0.04	0.08	1.0	1.0	1.0	1.0	1.0	1.0
	0.16	1.0	1.0	1.0	1.0	1.0	1.0

Table S3: Probability of tumor detection over time in the exponential growth model. The birth probability of the resident and mutant cells are given by $\frac{1}{2}(1+s_0)$ and $\frac{1}{2}(1+s_1)$, respectively. A higher growth coefficient of the mutant, s_1, can accelerate tumor progression. When $s_0 = s_1$, the detection time is independent of the mutation rate. The simulation results are averages over 10^7 runs. Parameter values: detection size $M = 10^9$ cells, driver mutation rate $u = 10^{-3}$, average cell division time is 3 days. (The value 0.0 corresponds to a probability below 10^{-3}.)

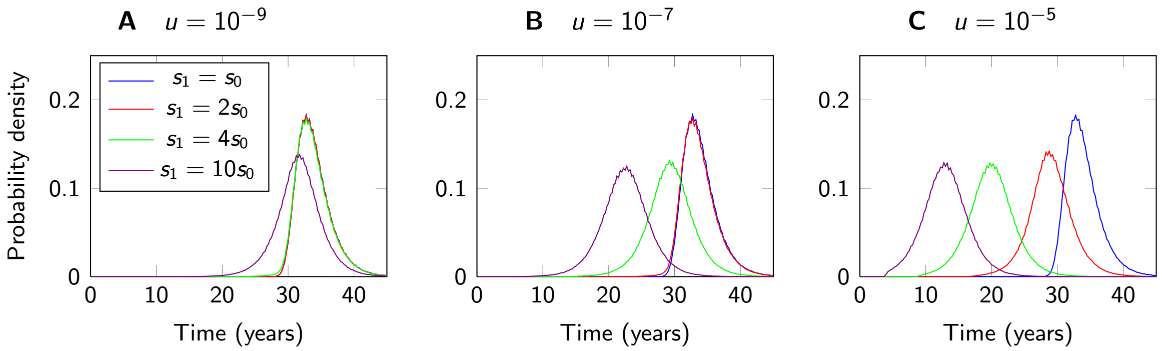

Fig. S1: Probability density of the tumor detection time in the exponential growth model. Effect on the distribution of the tumor detection time for different driver mutation rates and growth coefficients of the additional driver mutation. In panel A ($u = 10^{-9}$) only when $s_1 = 10s_0$ (purple line) tumor progression is significantly accelerated. Otherwise when $s_1 = 2s_0$ (red line) and $s_1 = 4s_0$ (red line) there is no effect on the tumor detection time (see panel A). If the mutation rate is increased to $u = 10^{-5}$ even when $s_1 = 2s_0$ (red line) affects the detection time (see panel C). The simulation results are averages over 10^7 runs. Parameter values: growth coefficient $s_0 = 0.004$, tumor detection size $M = 10^9$, average cell division time $T = 3$ days.

		Probability of detection after					
s_0	K	10 years	20 years	30 years	40 years	50 years	60 years
	10^4	0.0	0.0	0.0	0.0	0.0	0.0
0.002	10^6	0.0	0.0	0.0	0.0	0.0	0.0
	10^8	0.0	0.0	0.0	0.0	0.0	0.0
	10^4	0.0	0.0	0.0	0.0	0.0	0.0
0.004	10^6	0.0	0.0	0.0	0.0	0.0	0.003
	10^8	0.0	0.0	0.0	0.0	0.001	0.043
	10^4	0.0	0.0	0.0	0.0	0.0	0.001
0.01	10^6	0.0	0.0	0.008	0.02	0.032	0.044
	10^8	0.0	0.0	0.332	0.798	0.94	0.982
	10^4	0.0	0.0	0.0	0.001	0.001	0.001
0.02	10^6	0.0	0.019	0.042	0.065	0.087	0.108
	10^8	0.0	0.762	0.978	0.998	1.0	1.0
	10^4	0.0	0.001	0.001	0.002	0.002	0.003
0.04	10^6	0.017	0.062	0.105	0.146	0.185	0.222
	10^8	0.713	0.997	1.0	1.0	1.0	1.0

Table S4: Probability of tumor detection over time in the logistic growth model. The resident cells have a birth probability of $\frac{1}{2}(1+s_0(1-{}^{X}/_{K}))$ which depends on the current tumor size X. The birth probability of the mutant cells is constant $\frac{1}{2}(1+s_1)$. If the carrying capacity K is low but the mutation rate u is high (more precisely, if $Ku > {}^{s_0}/_{s_1}$), tumor progression is not decelerated. The simulation results are averages over 10^7 runs. Parameter values: growth coefficient $s_1 = s_0$, driver mutation rate $u = 10^{-9}$, detection size $M = 10^9$ cells, average cell division time is 3 days. (The value 0.0 corresponds to a probability below 10^{-3}.)

s_0	K	Probability of detection after 10 years	20 years	30 years	40 years	50 years	60 years
0.002	10^4	0.0	0.0	0.0	0.0	0.0	0.044
	10^6	0.0	0.0	0.0	0.0	0.0	0.114
	10^8	0.0	0.0	0.0	0.0	0.0	0.15
0.004	10^4	0.0	0.0	0.001	0.919	1.0	1.0
	10^6	0.0	0.0	0.003	0.962	1.0	1.0
	10^8	0.0	0.0	0.009	0.968	1.0	1.0
0.01	10^4	0.0	0.997	1.0	1.0	1.0	1.0
	10^6	0.0	0.999	1.0	1.0	1.0	1.0
	10^8	0.0	0.999	1.0	1.0	1.0	1.0
0.02	10^4	0.984	1.0	1.0	1.0	1.0	1.0
	10^6	0.994	1.0	1.0	1.0	1.0	1.0
	10^8	0.996	1.0	1.0	1.0	1.0	1.0
0.04	10^4	1.0	1.0	1.0	1.0	1.0	1.0
	10^6	1.0	1.0	1.0	1.0	1.0	1.0
	10^8	1.0	1.0	1.0	1.0	1.0	1.0

Table S5: Probability of tumor detection over time in the logistic growth model. The resident cells have a birth probability of $\frac{1}{2}(1+s_0(1-X/K))$ which depends on the current tumor size X. The birth probability of the mutant cells is constant $\frac{1}{2}(1+s_1)$. If the carrying capacity K is low but the mutation rate u is high (more precisely, if $Ku > s_0/s_1$), tumor progression is not decelerated. The simulation results are averages over 10^7 runs. Parameter values: growth coefficient $s_1 = s_0$, driver mutation rate $u = 10^{-3}$, detection size $M = 10^9$ cells, average cell division time is 3 days. (The value 0.0 corresponds to a probability below 10^{-3}.)

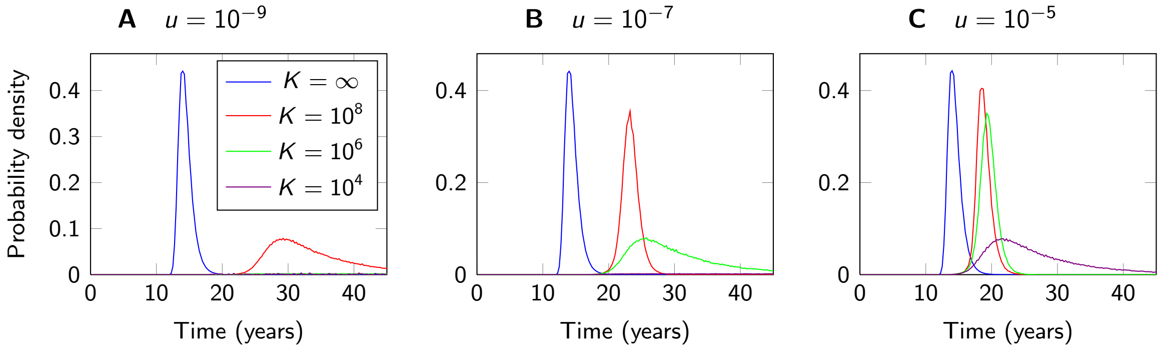

Fig. S2: Probability density of the tumor detection time in the logistic growth model. Effect on the distribution of the tumor detection time for different driver mutation rates and carrying capacities of the resident cells. If $Ku < s_0/s_1$, the carrying capacity can tremendously decelerate tumor progression. In panel A ($u = 10^{-9}$) no tumor can be detected within 50 years when $K \leq 10^6$ (green and purple line). The simulation results are averages over 10^7 runs. Parameter values: growth coefficient $s_0 = s_1 = 0.01$, tumor detection size $M = 10^9$, average cell division time $T = 3$ days.
