# Supplementary figures and images for: The effect of one additional driver mutation on tumor progression
Source: Evol Appl. 2012 Dec 10;6(1):34–45. doi: 10.1111/eva.12020 (PMC3567469; doi:10.1111/eva.12020)

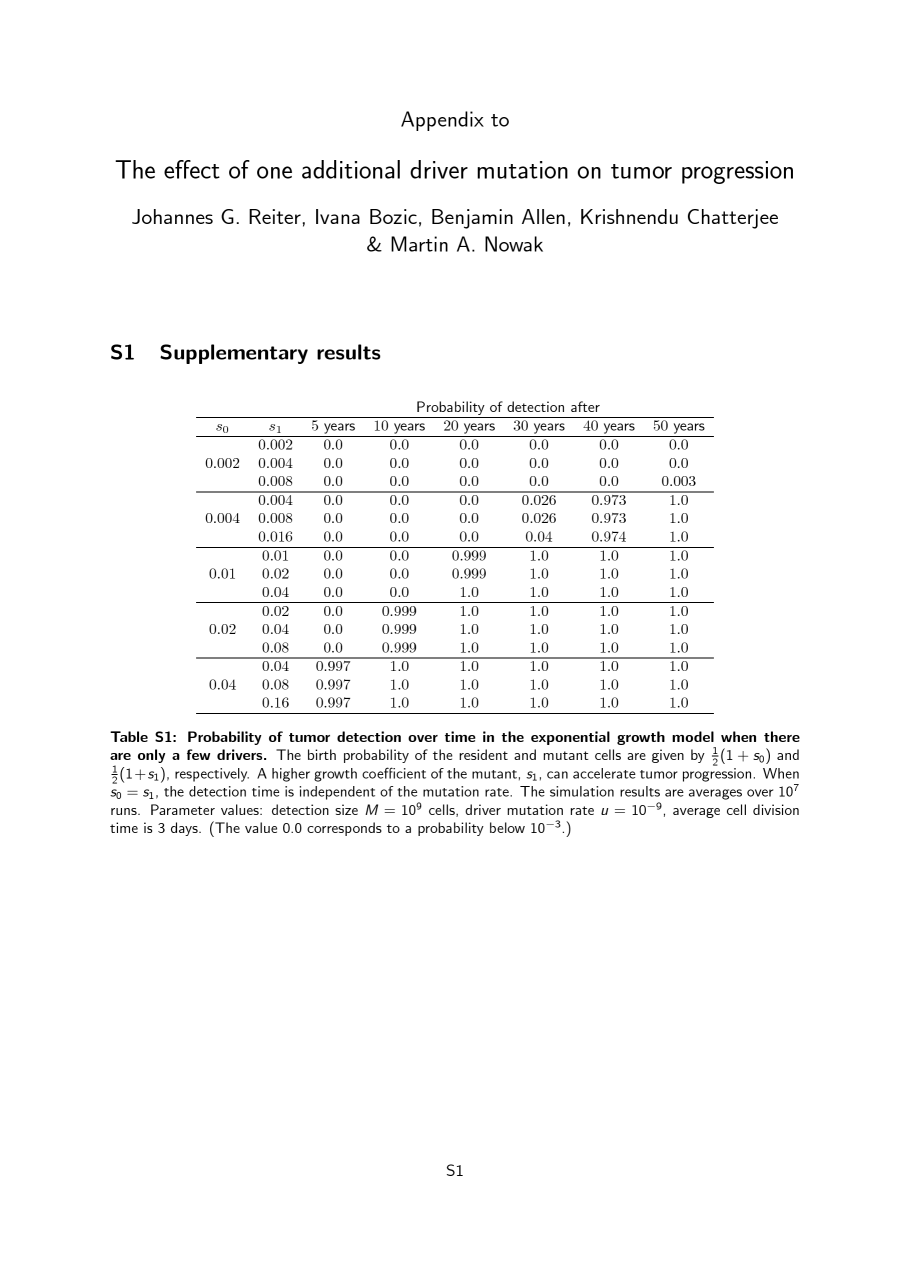

Supplement: Supplementary file 2 [file eva0006-0034-SD2.png]
